# Familial Mediterranean Fever and the Gut Microbiota: A Dual Perspective Review of Current Evidence

**DOI:** 10.31138/mjr.010725.flj

**Published:** 2026-06-01

**Authors:** Isabelle Basbouss-Serhal, Fatima Fayad

**Affiliations:** Molecular Biology Department, Sibline Governmental Hospital, Lebanon

**Keywords:** Familial Mediterranean Fever, autoinflammatory diseases, gut microbiome, colchicine, probiotics, dysbiosis

## Abstract

Familial Mediterranean Fever is a well-known autoinflammatory disease resulting from mutations in the MEFV gene. A recent development has linked FMF pathogenesis and mode of expression to the gut micro-biota. There may be a change in the gut microbiota profile of FMF patients, characterised by low diversity and a depletion of beneficial bacteria. Dysbiosis tends to be linked to increased gut permeability, systemic inflammation, and low response to colchicine treatment. Probiotics and prebiotics, in this case, may help restore the previous idyllic state of the microbial balance, along with a reduction in inflammatory markers, thereby demonstrating therapeutic merit. Notably, however, it did argue in some instances that changes in the microbiota were secondary to the genetic and inflammatory nature of FMF itself. It is still important to carry out longitudinal studies of naïve patients that will integrate metagenomics with immune profiling to ascertain whether microbial changes arise from causes, contributions, or coincidence in the pathogenesis of FMF.

## INTRODUCTION

Familial Mediterranean Fever (FMF) is the oldest and most common autoinflammatory disease. It is estimated that there are approximately 150,000 individuals affected by FMF globally, rendering it the most prevalent autoinflammatory disease across the world.^1^ FMF is more prevalent among individuals of Jewish, Armenian, Turkish, North African, and Arab heritage residing in the Mediterranean region. Additionally, instances of FMF have been reported in various other populations, including Greeks, Italians, and even Japanese.^2^ Familial Mediterranean fever (FMF) is caused by mutations in the MEFV gene,^3^ which encodes the protein pyrin. Certain MEFV variants are recognised as distinctly pathogenic; however, numerous variants are prevalent within the general population, and others remain of uncertain significance regarding their role in disease causation.^4^ It is a genetic periodic fever condition characterised by self-limited episodes of fever and polyserositis, with significant long-term consequences, such as amyloidosis.^5^ FMF’s most serious complication, AA amyloidosis, is characterised by extracellular deposits of an amorphous material containing an amyloidogenic derivative of the serum amyloid-associated (SAA) protein.^6^

Since 1972, colchicine has served as the standard therapy for Familial Mediterranean Fever (FMF),^7^ aiming to avert both sudden episodes and ongoing inflammation in all individuals, except a minor percentage of FMF patients who do not respond to the medication.^8^ Numerous individuals diagnosed with Familial Mediterranean Fever (FMF) recount experiencing their initial episode during early childhood, specifically before the age of 10 (65%) and 20 years (90%). Nonetheless, the timing of the first attack may vary based on genetic penetrance and phenotypic traits, with instances of onset occurring in individuals over the age of 50,^2^ including a notable case diagnosed at 86 years of age.^9^

The gastrointestinal tract evolves into a natural ecosystem that accommodates a diverse polymicrobial community, known as microbiota. This microbiota can undergo significant alterations under both healthy conditions and various diseases.^10^

The gut microbiota plays a crucial role in energy and vitamin metabolism, as well as pathogen protection, through the production and processing of bacterial components and metabolites.^11^ Additionally, it plays a crucial role in various aspects of human health and development, including the development and function of the immune system.^12^ *Bacteroides* and *Firmicutes* are the most common phyla in health, with *Spirochaetes*, *Proteobacteria*,^13–14^ *Verrucomicrobia*, *Actinobacteria*, *Lentisphaerae*, and *Fusobacteria* appearing in modest numbers.^15–16^ Various microbial ecosystem changes depend on pH in the intestinal lumen, redox potential, the type and amount of nutrients, gastrointestinal motility, and secretions.^16^ The precise function of intestinal microbiota in various conditions marked by recurrent genetically driven autoinflammatory diseases remains to be thoroughly elucidated. The gut microbiota plays a crucial role in human physiology and health, as well as in the preservation of systemic homeostasis. Among its various functions, it is essential for the development and regulation of the immune system.^17^ Indeed, numerous pathological conditions have been associated with changes in the quantity and/or composition of the microbiota. The therapeutic modulation of gut microbiota represents a potentially significant and novel approach, especially in the context of diseases marked by a chronic inflammatory condition.

Considering genetic and phenotypic traits, FMF patients serve as a compelling model for evaluating the influence of the genome and gene-environment interactions on the composition of gut microbiota. Recent findings in FMF indicate that the intestinal microbiota may play a significant role in the pathogenesis and severity of the disease.^18^

This article explores the relationship between the composition of the gut microbiota and the pathogenesis or severity of FMF, discussing two hypotheses: the direct involvement of alterations in the gut microbiota in the onset or flares of FMF, and the secondary effects due to inflammation, therapy, or lifestyle factors, and proposing future research directions.

## METHODS

A systematic search of the literature was also conducted on PubMed/Medline, Scopus, Web of Science, and DOAJ from inception until June 2025, using MeSH terms and FMF, microbiota, and relevant interventions as keywords. Original articles, reviews, and clinical trials written in the English language were considered for inclusion, while animal studies only, conference abstracts, and those without full text were not. Two reviewers independently screened titles, abstracts, and full texts, and the methodological quality was assessed using the Newcastle-Ottawa Scale and Cochrane Risk of Bias tool. Extracted data were study design, cohort size, microbial taxa, and clinical or mechanistic outcomes. The summaries of the included studies are presented in **Table 1**.

**Table 1.** Key studies on Familial Mediterranean Fever and gut microbiota: study design, population, and main findings.

**Author (Year)**	**Study Type**	**Population**	**Main Findings**	**Limitations**
**Ozen & Bilginer (2014)**	Review	N/A	Clinical overview of FMF and related autoinflammatory diseases	Narrative review; no new data
**Ben-Chetrit &Touitou (2009)**	Review / Epidemiology	Global FMF population	FMF prevalence, genetics, and phenotype distribution worldwide	Broad epidemiology; may lack recent updates
**Chae et al. (2011)**	Animal study	Mouse model	Pyrin gain-of-function mutations → IL-1β activation independent of NLRP3; severe autoinflammation	Animal model; may not fully translate to humans
**Van Gorp et al. (2020)**	Clinical study	FMF patient blood samples	Blood-based test for diagnosis and functional subtyping of FMF	Needs external validation; small sample
**Zemer et al. (1974)**	Clinical trial	FMF patients	Colchicine prevents FMF attacks	Early study; small cohort
**Lidar et al. (2004)**	Observational / Clinical	FMF patients nonresponsive to colchicine	Clinical, genetic, pharmacokinetic, socioeconomic factors in nonresponse	Small sample; heterogeneous population
**Deshayes et al. (2018)**	Cross-sectional	60 FMF patients	FMF and AA amyloidosis associated with gut dysbiosis	Single centre; cross-sectional
**Di Ciaula et al. (2020)**	Clinical / Microbiota	FMF patients	Gut microbiota influenced by environment and genetics	Observational; cannot establish causality
**Türkyılmaz et al. (2020)**	Observational	Children with FMF (attack-free)	Altered gut microbiota composition during remission	Small paediatric cohort
**Delplanque et al. (2024)**	Clinical cohort	FMF patients	Gut microbiota correlates with genotype, phenotype, and colchicine response	Moderate sample; observational
**Mansueto et al. (2022)**	Narrative review	N/A	Genetics, diet, and gut microbiota interplay in FMF; dietary influences on severity	Narrative review; no experimental data
**Ktsoyan et al. (2013)**	Metabolomics study	FMF patients on colchicine	Colchicine does not normalise altered microbial long-chain fatty acids	Small cohort; metabolomics only

### Gut Microbiota Changes in Patients with FMF

During the extended co-evolution between hosts and their microbes, the symbionts residing in the gut have become integrated into normal human metabolism and physiology.^18–19^ The development and proper functioning of the immune system are many aspects of human health and growth, in which the microbiota has been found to have a significant influence.^12–20–21^ Numerous factors affect the delicate balance between our gut microbiota and its human host, a symbiosis that has been carefully fine-tuned through millennia of co-evolution. The optimal state of equilibrium between microbiota and host depends on a complex interaction of genetic predispositions and environmental exposures that have shaped this relationship over many generations. A wide range of factors, including changes in diet or lifestyle, exposure to medications like antibiotics, hormonal fluctuations related to development or illness, and even ethnicity, can all disturb this fragile balance. Growing evidence suggests a possible link between the microbiota and Familial Mediterranean Fever (FMF), characterised by shifts in the relative abundance of microbial species. Whether such disturbances to the microbiota are relevant in driving the phenotypic manifestations of FMF, especially concerning genetic background, remains to be further explored.

To begin, we examine the growing evidence linking Familial Mediterranean Fever (FMF) and the gut’s microscopic inhabitants. Dysbiosis refers to the disruption of the gut microbiota balance and is the pathological basis of various diseases.^22^ Specifically, shifts in the complex community of microorganisms living in the gastrointestinal tract may contribute to the development and severity of FMF. The tiny residents of the intestine appear to have a surprising influence on a disease once thought to be solely determined by genetics. While early research established correlations between FMF and gut microbe composition, more recent studies have produced less conclusive or contradictory results. This section offers a balanced assessment of such research to highlight the ongoing issues in definitively establishing the link between FMF and the microbiota. Some studies failed to replicate earlier findings and suggested alternative pathways worth investigating further. Overall, the body of evidence underscores the complexity of FMF’s origins and the need for further investigation to better understand the connections between intestinal flora profiles and disease pathogenesis.

### Comparison of gut microbiota in FMF patients vs. healthy controls

A well-functioning gut microbiome performs important functions for the host. The importance of the gut microbiome in biology can be appreciated from the early stages of life. Postnatal developments in gut microbiota shape the neonatal immune system.^23–24^ Thereafter, it continues to play a significant role in various physiological processes, including the maintenance of homeostasis, immune regulation, and modulation of the central nervous system (CNS) and the enteric nervous system (ENS).^25–26^

Short-chain fatty acids (SCFAs), produced through organic fermentation by the human gut microbiota from dietary fibre that humans cannot digest, serve multiple functions in the body’s physiological processes, which have significant impacts on human health and disease.^27^ In FMF, several studies consistently report a decline in SCFA-producing bacteria, particularly *Faecalibacterium prausnitzii* and *Bifidobacterium spp*** .,** compared to healthy controls.^28^ *Faecalibacterium prausnitzii* (10% in healthy controls vs 4% in FMF patients), *Bifidobacterium* spp. (7% vs 3%),^28^ *Ruminococcus* spp., and *Roseburia* spp., compared to healthy controls.^29^ The loss of barrier integrity may allow lipopolysaccharides (LPS) and other microbial by-products to enter systemic circulation, activating the pyrin inflammasome and triggering FMF attacks.^30^ Activation of the pyrin inflammasome is a fascinating immune strategy used by the innate immune system to eliminate invading pathogens effectively.^31^

**Figure 1.**
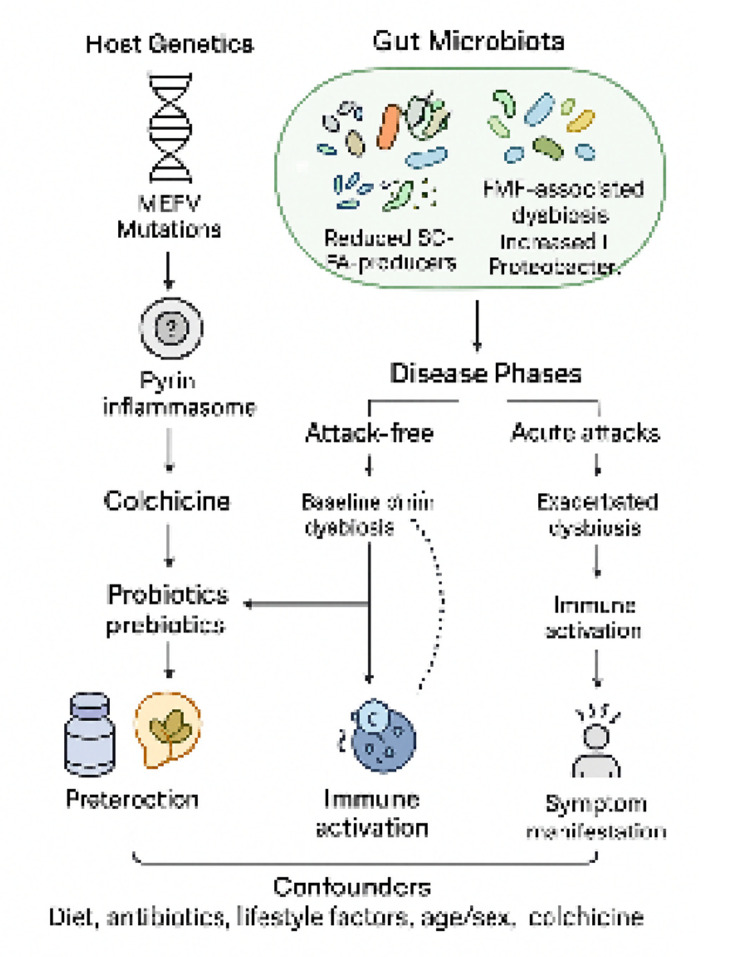
Interplay between host genetics, gut microbiota, and disease phases in Familial Mediterranean Fever.

Conversely, FMF patients tend to have a relative excess of Proteobacteria, such as *Enterobacteriaceae chu*, including *Escherichia coli* (4% in controls vs 12% in FMF),^29^ which are known for their pro-inflammatory potential and endotoxin production.^30–31^ Reduced levels of *Lactobacillus spp*. (5% vs 2%),^29^ and *Akkermansia muciniphila,* another protective bacterial group,^29^ have also been documented in FMF patients, reflecting the loss of microbes with immunomodulatory activity.^28–33^ Recent findings also suggest that the extent of these changes may depend on disease severity, MEFV mutation patterns, and colchicine therapy, with more severe genotypes showing greater dysbiosis.^34^ The overall framework of these microbiota-host interactions during FMF flares and remission is summarised in **Figure 1**.

Collectively, these comparative findings highlight that not only the diversity but also the qualitative composition of gut microbiota in FMF patients is shifted toward a pro-inflammatory state, which can fuel systemic inflammation and increase the risk of recurrent attacks.

### Microbiota shifts before, during, and after FMF attacks

As described above, baseline dysbiosis characterised by reduced SCFA-producing bacteria and increased pro-inflammatory taxa may be further exacerbated during FMF attacks, contributing to heightened systemic inflammation. Gut microbiota effects that would change dynamically during various phases of FMF would suggest an association of microbial composition with disease activity. Although so far scant evidence exists, it seems that the gut microbiota could undergo compositional changes before, during, or after FMF attacks, possibly interfering with immune activation and inflammatory response.^28–32^ During attack-free periods, FMF patients may display less microbial diversity and a changed relative abundance of some major taxa compared to healthy individuals. Importantly, decreased presence has been reported for the beneficial genera, including *Faecalibacterium* and *Bifidobacterium*, which produce anti-inflammatory short-chain fatty acids (SCFAs).^28^ The reduction of these SCFA-producing microbes may contribute to a continuous state of subclinical inflammation, preconditioning the host for episodes of inflammation. This type of evidence has also been supported by Di Ciaula et al. (2020) in accentuating the intersection of genetic susceptibility and microbial dysbiosis in the pathogenesis and modulation of FMF symptoms.

During acute FMF attacks, the gut microbiota shifts toward more pro-inflammatory characteristics, with intermittent increases in *Proteobacteria* and other endotoxin-producing bacteria. These changes likely intensify systemic inflammation, increase intestinal permeability, and activate innate immune pathways, including the pyrin inflammasome and IL-1β secretion.^35–36^ After attacks, microbiota may not fully revert to baseline, reflecting persistent dysbiosis.^28^ Metabolites derived from the microbiota, such as SCFAs, LPS, and bile acids, may directly modulate these pathways, influencing inflammasome priming and cytokine production**.** After attacks, microbiota may not fully revert to baseline, reflecting persistent dysbiosis.^28^

### Confounders in Microbiota-FMF Research

Interpretation of variation in gut microbiota in FMF patients is complicated by a number of possible confounders.^37^ Diets significantly influence the structure of microbial communities and the production of metabolites like SCFAs and bile acids. Fiber, fat, and protein variability may thus obscure disease-specific microbial signatures.

Colchicine therapy, the standard treatment for FMF, can alter gut microbiota diversity and composition irrespective of disease activity, both on beneficial and inflammatory taxa.^38^ Similarly, antibiotic exposure, both recent and chronic, can lead to transient or long-lasting changes in microbial populations, which makes comparison between FMF patients and healthy controls challenging.

Other potential confounders for the results are age, sex, ethnicity, comorbidities, and lifestyle factors (e.g., smoking, alcohol use, exercise), all of which can affect the microbiota. Over- or underestimation of microbiota differences in FMF may result from the exclusion of these variables.

Follow-up studies would need to have adequately matched control groups, collect detailed medication and diet histories, and, where possible, use treatment-naïve, recently diagnosed patients to control for confounding variables.^37–38^ This will help establish whether subsequent microbial alterations are causative, contributory, or secondary to FMF pathophysiology.

### Role of probiotics/prebiotics in FMF symptom control

The latest addition to the treatment modalities of familial Mediterranean fever (FMF) is probiotics and prebiotics.^34^ However, current evidence is largely preliminary, and randomised controlled trials are lacking; thus, therapeutic recommendations should be interpreted cautiously. FMF treatment combined both the modulation of gut microbiota and the systemic inflammation countermeasure. It is now also associated with abnormal gut dysbiosis in the form of reduced microbial diversity and striking depletion of critical anti-inflammatory bacterial taxa such as *Faecalibacterium prausnitzii* and *Bifidobacterium spp*.^28–33^ Clinical intervention of the microbiota is expected to bring about some recovery of the abnormality in microbial flora. Probiotics are live microorganisms believed to bring health benefits to their host; they may improve gut barrier function, reduce endotoxin translocation, and possibly inhibit the activation of the pyrin inflammasome major pathology of FMF. In FMF patients, supplementation with *Lactobacillus acidophilus* INMIA 9602 has been shown to modulate gut microbiota, reduce Candida colonisation, and potentially lower systemic inflammatory markers.^39^ Additionally, studies in motion-related autoinflammation and autoimmunity found that relevant probiotic supplementation would have reduced levels of high inflammatory markers, interleukin-1β (IL-1β), as well as C-reactive protein (CRP). Since causative FMF flares enhance both IL-1β and CRP levels, the use of probiotics is alluded to as having some curative value during FMF-related flares.^28–30,40^ These improvements might encourage further microbiota-targeted therapeutic explorations, with a new hope being generated for FMF therapy.

Current clinical trials focused on Familial Mediterranean Fever (FMF) are not providing enough evidence as of now. However, preliminary findings indicate that probiotic supplementation could diminish the frequency of attacks and relieve gastrointestinal symptoms in patients intolerant or resistant to colchicine therapy. Some specific pilot studies have looked into the benefits of *Lactobacillus* and *Bifidobacterium* strains, suggesting potential immunomodulating effects and general improvements in health in FMF patients.^30^ And, meanwhile, prebiotics, that is to say, non-digestible fibre such as inulin and fructo-oligosaccharides, have been proven effective in promoting beneficial house non-harmful anti-inflammatory microbes in the intestines and stimulating the production of short-chain fatty acids (SCFAs), including butyrate. SCFAs are immunoregulatory ones, strengthening epithelial barrier integrity and suppressing the production of proinflammatory cytokines. Together, microbiota-targeted interventions may help restore gut homeostasis, reduce systemic inflammation, and potentially allow for lower colchicine doses in FMF management.

### Effects of colchicine on gut microbial diversity

Colchicine, the mainstay treatment for Familial Mediterranean Fever (FMF), is now known to affect gut microbiome diversity, which affects the control of disease and tolerance in the gastrointestinal tract.^40^ As a microtubule-disrupting agent, it affects not only the migration of inflammatory cells and the release of cytokines but also gastrointestinal physiology, including epithelial cell turnover and mucosal immunity.^43^ These alterations may indirectly influence the gut microbiota’s composition and function. According to a study by Ozen et al. (2017), FMF patients on long-term colchicine treatment showed decreased microbial diversity and considerable changes in the abundance of key bacterial taxa compared to healthy controls. While this dysbiosis could be a partial reflection of inflammation related to the disease, colchicine may also intervene in the gut ecosystem by decreasing beneficial anaerobic bacteria such as *Faecalibacterium* and *Bifidobacterium*, which are otherwise known to have anti-inflammatory and gut-protective actions.^30^ High doses of colchicine are often associated with gastrointestinal side effects (for example, diarrhoea and abdominal pain),^44^ which may also be partially consequent to alterations in the gut microbiota. It is increasingly being recorded whether the anti-inflammatory effects of colchicine would have been partly through microbiota–immune system interactions and whether microbiota-targeted therapies (for example, probiotics and prebiotics) might increase the responsiveness of colchicine or lessen its side effects.

### Relationship Between Microbiota Composition and MEFV Mutations

Recent data suggest that the gut microbiota composition of FMF patients could be related to such factors as mutation type and severity in MEFV gene mutations, hinting at the interaction between host genetics and microbial ecology. The MEFV gene encodes pyrin, an important negative regulator of the inflammasome complex, and mutations in this gene—particularly homozygous or compound heterozygous mutations such as M694V/M694V—have been related to more severe disease phenotype, increased IL-1β activity, and greater attack frequency.^34^ A few reports suggest that these genetic variants may influence the host immune environment in ways that alter gut microbiota by changing either mucosal immune responses or permeability of the gut barrier. Delplanque M et al. (2024) reported significant changes in microbial diversity and composition among FMF patients, although they did not categorise their data specifically according to MEFV mutation type. The recent microbiome-genotype studies in other autoinflammatory conditions have revealed that individuals carrying more severe inflammatory genotypes are often enriched for pro-inflammatory bacterial taxa (e.g., *Enterobacteriaceae* and *Proteobacteria*) and depleted of short-chain fatty acid (SCFA)-producing commensals (e.g., *Faecalibacterium prausnitzii*). Following this line of reasoning, it is therefore possible that FMF patients harbouring a more severe MEFV mutation may have a more dysbiotic microbiota that could enhance the inflammasome and contribute to persistent subclinical inflammation. The emerging association thus provides the opportunity for host-microbiome interplay studies to explore microbial diagnostics linked to genotype-specific disease severity and offer pathways for developing personalised MEFV mutation-profile-based microbiota-modulating therapeutics.

### Studies Reporting Limited or No Clear Relationship Between FMF and Gut Microbiota

However, the involvement of gut microbiota in familial Mediterranean fever (FMF) is an active area of investigation, and available evidence does not yet definitely demonstrate causality. Mutations of the MEFV gene represent the predominant cause of FMF, resulting in innate dysfunction of the pyrin inflammasome and thereby leading to excessive production of inflammatory cytokines, including IL-1β, independent of microbial stimulation.^43 45^ Cohort and pilot studies have failed to show statistically significant differences in gut micro-biota composition or diversity when FMF patients were compared with healthy controls; confounding variables such as diet, colchicine treatment, and antibiotic use were accounted for.^46–47^ Taken together, these findings indicate that the alterations in the gut microbiota observed may act secondarily to systemic inflammation or drug effects rather than serving as agents triggering the FMF pathogenesis.

Environmental factors, such as lifestyle and diet,^48^ as well as under the influence of colchicine therapy,^38^ can already greatly shape the gut microbial profiles and might obscure any microbial changes specific to FMF. Other confounders, including co-infections or unrelated gastrointestinal disorders, may further mask any microbial signature associated with FMF. Experimental studies on inflammasome activation clearly state the intrinsic cellular mechanisms, such as the pyrin mutation, triggering FMF attacks, where a microbial trigger is not necessarily required. This supports the notion of considering dysbiosis of gut microbiota, if there is any, as an epiphenomenon or secondary thing rather than the cause of FMF.^43–44^

Because of FMF’s chronic genetic characteristics, the observed changes in the microbiota may be incidental, compensatory, or indicative of inflammatory activity rather than inducing inflammatory activity. The consistent activation of the inflammasome in FMF implies that the pathogenic process is self-sufficient and independent of the microbial environment. Given the conflicting evidence, along with major environmental and therapeutic confounding factors, no robust conclusions point toward substantial involvement of microbiota. Hence, larger controlled, prospective studies on newly diagnosed and treatment-naive FMF patients are required to clarify whether gut microbiota are causative, synergistic, or conveniently indifferent to FMF pathogenesis.

## DISCUSSION

This review synthesises the comprehensive picture of interaction between Familial Mediterranean Fever (FMF) and gut microbiota, combining the current evidence on microbial profile, functional metabolites such as short-chain fatty acids (SCFAs), activation of inflammasomes, and plausible therapeutic intervention. Compared with prior articles in the Mediterranean Journal of Rheumatology, our review synthesises a broader aggregation of mechanistic and clinical evidence that relates the dysregulation of gut microbiota to the pathophysiology of FMF and the designation of putative intervention targets.

Clinically, these microbial changes could be used to guide new approaches to disease monitoring and management, such as dietary modulation, probiotics or prebiotics, and colchicine optimisation. Furthermore, the identification of microbiota-based biomarkers would also be useful for determining disease activity or predicting treatment outcome.

While several studies document concordances between microbial dysbiosis and FMF, limitations including small case numbers, methodological heterogeneity, and a lack of randomised controlled trials for probiotics or prebiotics remain. These limitations emphasise the need for high-quality mechanistic and clinical research to explore potential causal associations and therapeutic possibilities.

Overall, this review not only summarises what is known and proposes an agenda for future research but also emphasises the incorporation of microbiota analysis into clinical care in FMF and calls for the investigation of personalised interventions.

## CONCLUSION

That is, future studies designed to prove beyond doubt the role of gut microbiota in FMF should include longitudinal studies on newly diagnosed, treatment-naïve patients. Also, these studies must control for important confounding factors affecting microbiota composition: diet, colchicine therapy, antibiotic treatment, and lifestyle factors, among others. Potentially, high-resolution metagenomics sequencing employed along with metabolomics/immunological settings during attack and remission phases can inform on the full microbial dynamics, which would also shed light on micro-biota-host interactions. Causality could be inferred in experimental setups involving faecal microbiota transplantation into germ-free animal models, which are required to prove that microbiota from FMF patients can elicit inflammatory phenotypes. This integrative plan may well elucidate whether alterations of gut microbiota are drivers, modifiers, or mere bystanders in FMF pathogenesis, thereby directing the search for new microbiota-directed therapies.
